# Impact of social media on pregnancy in India

**DOI:** 10.6026/973206300212891

**Published:** 2025-08-31

**Authors:** Rajlakshmi M., P. Minthami Sharon, Jasmine Kavitha Washington, Sindhu R.S.S

**Affiliations:** 1 Department of Obstetrics and Gynaecology, Sree Balaji Medical College and Hospital, Chennai, India

**Keywords:** Pregnancy, social media, public health, mental health, anxiety

## Abstract

Pregnancy is a transformative period that may be influenced by digital engagement. This cross-sectional study among 96 pregnant women assessed the impact of demographic and obstetric factors on perceptions of social media and its link to anxiety. Higher education and later trimester were significantly associated with more positive social media experiences. However, increased social media engagement correlated with heightened pregnancy-related anxiety (p < 0.001). Thus, we show the dual impact of social media and the importance of incorporating digital literacy and emotional support into antenatal care.

## Background:

Pregnancy is a phase marked by physiological and emotional transitions that may increase psychological vulnerability [1]. In recent years, the proliferation of digital technologies has transformed the sources from which expectant mothers seek information and support [2]. Among these, social media has emerged as a prominent medium through which pregnant women access health content and community advice [3]. Platforms such as Facebook, Instagram, YouTube and TikTok offer a range of maternal content, including birth narratives, expert recommendations and peer experiences [4]. Expectant women increasingly engage with these platforms through influencer followership, online support groups and parenting forums, particularly among digitally literate users [5]. While digital engagement offers opportunities for health information sharing, it also carries risks. In the Indian context, social media often promotes idealized and unrealistic portrayals of pregnancy and motherhood, which may contribute to heightened anxiety, emotional distress, and negative self-perceptions among expectant mothers [6]. Exposure to curated content and social comparisons may heighten anxiety and diminish maternal confidence [7]. Additionally, the reliance on social media for medical guidance raises concerns about misinformation and the variable credibility of online sources [8]. A study affirm the value of social media as a source of reassurance and peer validation during pregnancy [9], while others highlight its association with increased anxiety, especially among first-time mothers and during early gestation [2, 7]. Despite this growing interest, much of the evidence is qualitative and robust quantitative research exploring these dynamics remains limited [10]. Furthermore, variables such as age, education, parity, or gestational trimester influence perceptions of social media or related emotional outcomes [10]. Therefore, it is of interest to address these gaps by quantitatively assessing the relationship between social media engagement and pregnancy-related anxiety and by evaluating how demographic and obstetric variables modulate these associations.

## Materials and Methods:

## Study design and setting:

A cross-sectional, descriptive, observational study was conducted over a period of three months in the Department of Obstetrics and Gynaecology at a tertiary care institution located in Chennai. The study aimed to explore the relationship between social media engagement and pregnancy-related anxiety among expectant mothers. Ethical clearance was obtained from the Institutional Ethics Committee prior to the initiation of the study. All participants provided written informed consent after being informed about the study's objectives, voluntary nature and confidentiality assurances.

## Study population and eligibility criteria:

During the three-month study period, a total of 96 eligible pregnant women who visited the antenatal outpatient department of Sree Balaji Medical College and Hospital were approached and enrolled, fulfilling the calculated sample size target through consecutive sampling. The study included 96 pregnant women aged 18 years and above. Inclusion criteria were: (1) pregnant women regardless of gestational age and (2) individuals actively using social media platforms-such as Facebook, Instagram, YouTube, or pregnancy-specific online forums-to seek or share pregnancy-related information. Exclusion criteria comprised: (1) women diagnosed with severe pre-existing mental health conditions that could confound anxiety measurements and (2) women with high-risk pregnancies necessitating extensive medical intervention, as their experiences may not reflect those of the general pregnant population (Table 1 - see PDF).

## Procedure and data collection:

Data were collected using a predesigned, semi-structured, self-administered questionnaire. Eligible participants were recruited from the antenatal outpatient clinic at the study site. Initial screening was conducted using the predefined inclusion and exclusion criteria. Eligible individuals were informed about the research objectives, procedures, confidentiality protocols and their right to withdraw at any time without affecting their clinical care. Following informed consent, participants completed the questionnaire either during their hospital visit or online via a secure link. The questionnaire comprised four key sections. The first section collected demographic details such as age, education level and occupational status. The second section captured obstetric history, including parity and current trimester. The third section assessed pregnancy-related anxiety using the Pregnancy-Related Anxiety Questionnaire - Revised 2 (PRAQ-R2), a validated 10-item scale that measures three core anxiety domains: (a) fear of childbirth, (b) concerns about fetal health, specifically regarding physical or mental disabilities and (c) worries related to body image [11]. Each item was rated on a five-point Likert scale ranging from "definitely not true" to "definitely true". The fourth section evaluated social media engagement through a modified version of the Social Media Engagement Questionnaire (SMEQ) [12], adapted to reflect usage patterns specific to pregnancy- and motherhood-related content. This section captured the frequency of engagement (e.g., number of days per week), types of content consumed (informational, emotional support, peer experiences) and the emotional responses triggered by such content (e.g., feeling reassured, neutral, or anxious).

## Ethical considerations:

Confidentiality and data security were strictly maintained throughout the study. All responses were anonymized before analysis and stored securely. Participation was entirely voluntary and subjects retained the right to withdraw from the study at any point without jeopardizing their access to clinical care.

## Statistical analysis:

Collected data were compiled and analyzed using IBM SPSS Statistics Version 22 (SPSS Inc., Chicago, IL). Descriptive statistics-including means, standard deviations, frequencies and percentages-were used to summarize demographic variables, obstetric details and social media engagement patterns. For pregnancy-related anxiety, total and subscale scores of the PRAQ-R2 were computed. The subscales included: fear of childbirth (3 items; score range: 3-15), concerns regarding fetal disability (4 items; range: 4-20) and body image worries (3 items; range: 3-15), with the total PRAQ-R2 score ranging from 10 to 50. Social media engagement was assessed by calculating the average frequency of weekly interactions with pregnancy-related content. Additionally, participants' emotional responses to such content were quantified. To examine relationships between social media engagement and demographic or obstetric characteristics, Chi-square tests were applied. All statistical tests were two-tailed and a p-value < 0.05 was considered statistically significant.

## Results:

Of the 96 pregnant women surveyed, 54 (56.3%) exhibited a positive perception of social media use for pregnancy-related information, while 42 (43.7%) had a negative perception. Among those with a positive perception, the highest proportion belonged to the 26-30 age group 20 (37.0%), followed by the 31-39 age group 18 (33.3%). In contrast, the negative perception group also had a sizable representation from the 26-30 age group 16 (38.1%), but a slightly higher proportion was seen in the younger 18-25 age group 14 (33.3%). Educational level showed a statistically significant association with perception (p = 0.042), where 34 (63.0%) women with university-level education or above had a positive perception, compared to only 18(42.9%) in the negative group. Occupation did not show a significant association (p = 0.274), though healthcare workers were more likely to have a positive perception 16 (29.6%) compared to only 8(19.0%) in the negative group (Table 2 - see PDF). Among the participants, parity and trimester of pregnancy showed notable trends in relation to their perceptions of social media use. Of the 54 women who had a positive perception, 36 (66.7%) were multigravida and 18 (33.3%) were primigravida. In contrast, among the 42 women with a negative perception, 22 (52.4%) were multigravida and 20 (47.6%) were primigravida. Although multigravidas appeared more likely to perceive social media positively, this difference was not statistically significant (p = 0.142). Regarding the trimester of pregnancy, a statistically significant association was observed (p = 0.018). Among those with a positive perception, 22 (40.7%) were in their third trimester, 20 (37.0%) in the second trimester and 12 (22.2%) in the first trimester. In contrast, among those with a negative perception, 20 (47.6%) were in their first trimester, 12 (28.6%) in the second trimester and only 10 (23.8%) in the third trimester (Table 3 - see PDF).

Table 4 (see PDF) shows 42 women (43.8%) reported high levels of pregnancy-related anxiety, while 54 women (56.2%) experienced low or no anxiety. A strong association was observed between social media behavior and anxiety levels (p < 0.001 across all parameters). Among those with low or no anxiety, a large majority-44 (81.5%)-regularly followed pregnancy or motherhood-related content, compared to only 12 (28.6%) among those with high anxiety. Similarly, 28 women (51.9%) in the low/no anxiety group actively engaged in pregnancy discussions on social media, while only 4 (9.5%) in the high anxiety group did so. The perception of pregnancy being positively influenced by social media was reported by 46 (85.2%) women with low anxiety, in contrast to just 14 (33.3%) in the high anxiety group. Furthermore, 40 (74.1%) low-anxiety participants felt reassured by pregnancy-related posts, compared to only 8 (19.0%) among those with high anxiety. Notably, 26 women (61.9%) in the high anxiety group reported that social media increased their anxiety, whereas this was true for only 10 (18.5%) of those with low or no anxiety (Table 4 - see PDF). Table 5 (see PDF) shows social media usage was found to have a significant association with specific domains of pregnancy-related anxiety as measured by the PRAQ-R2 scale. Of the 54 women identified as high social media users, 38 (70.3%) reported a high level of fear regarding giving birth, compared to only 16 (38.1%) among the 42 women with low or no social media use (p = 0.001). Similarly, 36 high users (66.7%) expressed significant worries about having a physically or mentally handicapped child, whereas this concern was present in only 14 (33.3%) of the low-use group (p < 0.001). Concerns related to physical appearance were also more prevalent among high social media users, with 39 women (72.2%) reporting anxiety in this domain, in contrast to just 17 women (40.5%) among low or non-users (p < 0.001).

## Discussion:

This study explored the nuanced role of social media in shaping pregnant women's perceptions and levels of pregnancy-related anxiety. The findings reveal a complex interplay between demographic characteristics, obstetric factors, digital engagement patterns and emotional well-being. While social media can offer accessible information and peer support, it also has the potential to amplify emotional distress and promote unrealistic expectations, particularly among vulnerable groups. A key finding was the significant association between higher education levels and positive perceptions of social media use. Among women with a university education or above, 63.0% reported a positive perception, in contrast to 42.9% of those with lower education levels (p = 0.042). This aligns with the observations of Skouteris and Savaglio (2021) [13], who found that educated women tend to engage more critically with social media, seeking trustworthy and practical health content during pregnancy. Their ability to distinguish credible information from misleading content likely enhances the benefits they derive from online platforms. In contrast, women in their first trimester were more likely to have a negative perception of social media (47.6%), compared to only 22.2% with a positive perception in the same group-a statistically significant difference (p = 0.018). This is consistent with the findings of Sanli *et al.* (2025), who noted that early pregnancy, being a phase of heightened emotional vulnerability and intense information seeking, often coincides with exposure to alarming or contradictory content online [14]. This may lead to confusion, heightened fear and emotional instability, particularly among those who are navigating pregnancy for the first time. Although the association between parity and perception was not statistically significant (p = 0.142), first-time mothers (primigravidae) tended to report more negative perceptions.

Social media behavior showed a marked relationship with anxiety outcomes. Among women with low or no anxiety, 81.5% regularly followed pregnancy-related content, 51.9% actively participated in discussions and 74.1% felt reassured by the posts they encountered. In contrast, among women with high anxiety, only 28.6% followed such content, 9.5% engaged in discussions and merely 19.0% felt reassured; moreover, 61.9% reported that social media increased their anxiety (all p-values < 0.001). These results resonate with Chee *et al.* (2023) [3], who described how curated influencer content can both support and harm-offering emotional solidarity for some, while causing self-comparison and insecurity for others. Similarly, Lowe-Calverley and Grieve (2021) [7], highlighted that idealized portrayals of pregnancy and motherhood can drive stress and feelings of inadequacy. Similarly, Rosenbaum *et al.* (2024) discussed how social media acts as a commercial gateway, often pushing unrealistic health and wellness expectations to pregnant women through influencers and sponsored content [15]. Additionally, this study found that high social media users exhibited significantly greater anxiety across three domains measured by the PRAQ-R2: fear of childbirth (70.3%), concern about having a handicapped child (66.7%) and body image issues (72.2%), compared to low or non-users (all p < 0.001).

Munro *et al.* and Sanders *et al.* similarly emphasized how misinformation online can shape decision-making around labor and pain management, increasing anxiety and influencing elective interventions like C-sections [16, 17]. Pregnancy-related anxiety among pregnant women in our study showed results comparable to those reported by Sanli *et al.* (2025) and Al Ghadeer *et al.* (2021), both conducted in similar settings [14, 18]. This study has certain limitations. First, its cross-sectional design restricts the ability to establish causality between social media use and pregnancy-related anxiety. Second, the relatively small sample size, drawn from a single tertiary care hospital in Chennai, may limit the generalizability of the findings to broader populations with different sociodemographic backgrounds. Additionally, self-reported data on social media usage and emotional responses may be subject to recall bias or social desirability bias. The exclusion of women with high-risk pregnancies or mental health disorders, while methodologically justified, may also have omitted important perspectives from the sample. Future research should explore longitudinal designs to better understand the temporal relationships between social media exposure and emotional changes during pregnancy. Digital literacy programs targeting pregnant women, especially those in early pregnancy or with limited education, could empower them to critically evaluate online content. Healthcare providers should consider integrating discussions around responsible social media use into routine antenatal care, helping women discern reliable sources and avoid anxiety-inducing content. Collaborative efforts with digital platforms and influencers may also be valuable in promoting accurate, supportive and evidence-based pregnancy information online.

## Conclusion:

The dual impact of social media on pregnant women's perceptions and emotional well-being is shown. While it offers reassurance and useful information-especially for educated women in later pregnancy-it also contributes to anxiety, particularly in first-time or early-stage mothers. High engagement was linked to fears about childbirth, baby's health and body image. Thus, we show the importance of integrating digital literacy into antenatal care, enabling women to navigate online content critically and protect their emotional health.

## Declaration on publication ethics:

The authors state that they adhere to COPE guidelines on publishing ethics as described elsewhere at https://publicationethics.org/. The authors also undertake that they are not associated with any other third party (governmental or non-governmental agencies) linking with any form of unethical issues related to this publication. The authors also declare that they are not withholding any information that is misleading to the publisher in regard to this article. There is no pre-print already published elsewhere for this article.

## Declaration on official E-mail:

The corresponding author declares that the lifetime official e-mail from their institution is not available for all authors.

## Ethical approval:

The study was approved by Institutional Human Ethics Committee of Sree Balaji Medical College and Hospital, Chennai, India.

## Funding:

No Funding
